# Phenotypic convergence of Menkes and Wilson disease

**DOI:** 10.1212/NXG.0000000000000119

**Published:** 2016-11-17

**Authors:** Boglarka Bansagi, David Lewis-Smith, Endre Pal, Jennifer Duff, Helen Griffin, Angela Pyle, Juliane S. Müller, Gabor Rudas, Zsuzsanna Aranyi, Hanns Lochmüller, Patrick F. Chinnery, Rita Horvath

**Affiliations:** From the John Walton Muscular Dystrophy Research Centre (B.B., D.L.-S., J.D., H.G., A.P., J.S.M., H.L., R.H.), and MRC Centre for Neuromuscular Diseases, Institute of Genetic Medicine Institute of Genetic Medicine, Newcastle University, UK; Department of Neurology (E.P.), University of Pecs, Hungary; MRI Research Centre (G.R.), and MTA-SE NAP B Peripheral Nervous System Research Group (Z.A.), Department of Neurology, Semmelweis University, Budapest, Hungary; MRC-Mitochondrial Biology Unit (P.F.C.), and Department of Clinical Neurosciences (P.F.C.), Cambridge Biomedical Campus, University of Cambridge, UK.

Menkes disease is an X-linked multisystem disorder with epilepsy, kinky hair, and neurodegeneration caused by mutations in the copper transporter *ATP7A*. Other *ATP7A* mutations have been linked to juvenile occipital horn syndrome and adult-onset hereditary motor neuropathy.^1,2^ About 5%–10% of the patients present with “atypical Menkes disease” characterized by longer survival, cerebellar ataxia, and developmental delay.^2^ The intracellular copper transport is regulated by 2 P type ATPase copper transporters ATP7A and ATP7B. These proteins are expressed in the trans-Golgi network that guides copper to intracellular compartments, and in copper excess, it relocates copper to the plasma membrane to pump it out from the cells.^3^ *ATP7B* mutations cause Wilson disease with dystonia, ataxia, tremor, and abnormal copper accumulation in the brain, liver, and other organs.^4^

Here, we report an *ATP7A* mutation, manifesting with an unusual complex phenotype resembling Wilson disease.

## Methods.

A 29-year-old man was born to a nonconsanguineous family; his father and paternal uncle suffer from genetically confirmed X-linked Kennedy disease. He achieved normal developmental milestones and manifested with progressive gait ataxia and proximal and distal leg weakness with early teens onset. Four limb spasticity evolved with extrapyramidal movement disorder, and he started using wheelchair at the age of 20. Clinical examination detected normal stature with no skeletal and joint changes and no connective tissue, cardiovascular, or hepatic abnormalities. He had normal vision and no evidence of Kayser-Fleischer rings, but bilateral nystagmus was present. He had severe spasticity and dystonia in all four extremities. Deep tendon reflexes were increased (4+) except for absent ankle jerks; clonus was present; and Babinski sign was positive. Cerebellar symptoms associated include intention tremor, dysmetria, and dysdiadochokinesis, and Romberg test was positive. His gait was spastic-ataxic (figure, A and C). He had dysarthria but preserved cognition and no mental illness. Routine laboratory investigations were normal. Metabolic tests including coeruloplasmin (0.19 g/L) and copper in serum and urine were repeatedly normal. EMG of the left tibial anterior muscle revealed increased insertional activity with fibrillations and larger motor units. Nerve conduction velocities were normal, but amplitudes were reduced in the peroneal and medial nerves, suggesting axonal motor neuropathy. Initial brain MRI at 9 years of age indicated high signal intensity of bilateral globus pallidus on T2-weighed images. Follow-up scan at age 29 years showed mildly increased signal intensity of bilateral globus pallidus on fluid-attenuated inversion recovery (FLAIR) sequences but not on T2-weighed images and mild cerebellar atrophy (figure, B).

**Figure:**
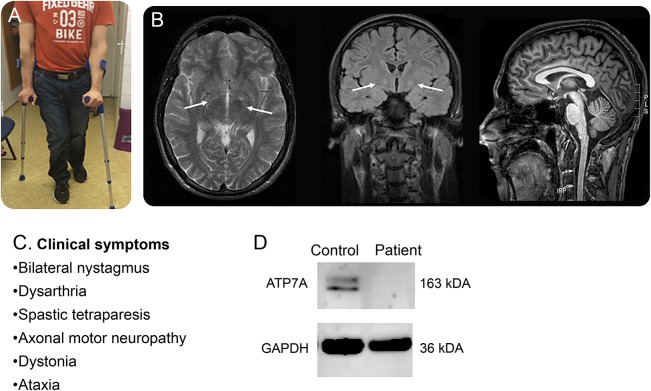
Clinical presentation, neuroimaging, and immunoblotting (A) Photograph of the patient illustrates spasticity. (B) Neuroimages indicate bilateral abnormal signal intensity in the globus pallidus (T2, fluid-attenuated inversion recovery) and mild cerebellar atrophy (T1). (C) Leading clinical symptoms. (D) Immunoblot analysis detected severely reduced ATP7A protein in the patient's fibroblasts.

Genetic testing was negative for Kennedy disease and common ataxias. Illumina TruSeq 62 Mb exome capture, sequencing (100 bp paired-end reads, HiSeq 2000; Illumina, San Diego, CA), and alignment (UCSC hg19) was performed in the patient. Potentially deleterious recessive or X-linked variants were identified using QIAGEN Ingenuity Variant Analysis and validated by Sanger sequencing. Immunoblotting was performed using standard protocols.^1^

## Results.

The patient carried the hemizygous c.2279A>G, p.(Tyr760Cys) variant in *ATP7A.* His healthy mother was heterozygous for the sequence change which was absent in her healthy brother. The variant was rare (Exome Aggregation Consortium: 4 in 87,766 heterozygous X chromosomes, no hemizygous), predicted highly deleterious by 5 different prediction tools, and affected a highly conserved residue in the third transmembrane domain of ATP7A. The neighboring p.(Ser761Pro) has been associated with the moderate Menkes phenotype.^2^ Immunoblotting confirmed severely reduced ATP7A protein in the patient's fibroblasts compared with the control (figure, D).

## Discussion.

We identified the c.2279A>G, p.(Tyr760Cys) *ATP7A* variant in a patient with complex neurologic signs of spastic tetraparesis, ataxia, dystonia, and axonal motor neuropathy. The mutation segregated with the disease in the family and resulted in reduced ATP7A protein. Smaller amounts of functional ATP7A have been reported as sufficient to cause milder phenotypes.^1^ However, the association of spastic tetraparesis, ataxia, dystonia, and axonal motor neuropathy observed in our patient is remarkably different from any of the phenotypes reported with mutations in *ATP7A*. Wilson disease presents with heterogeneous hepatic and/or neurologic presentation, including variable combinations of dystonia, cerebellar, extrapyramidal, or psychiatric symptoms.^4^ White matter lesions and cerebral atrophy are seen in mild Menkes disease, but T2-weighted high signal intensities, indicating abnormal copper deposition in the globus pallidus, are more characteristic for Wilson disease, a copper retention disorder caused by *ATP7B* mutations.^4^ *ATP7A* variants as modifiers have been studied in Wilson disease based on a recent canine model carrying mutations in either *ATP7A* or *ATP7B*.^5^ The 2 proteins share sequence homology for residues involved in copper translocation, regardless of their directionally different trafficking. A 38 amino acid segment within the third transmembrane domain is implicated in the trans-Golgi retention of ATP7A.^6^ This same region is mutated in our patient suggesting subsequent ATP7A mislocalisation and misfolding in the disease mechanism. It is possible that the mutation triggers conformational changes and induces aberrant protein-protein interactions leading to impaired ATP7A trafficking.^3^

Our case supports the large phenotypic variability of *ATP7A* mutations and highlights that deficiency of the two copper transporter ATPases may cause overlapping phenotypes. *ATP7A* seem to be a human disease gene with very variable clinical presentations, and better understanding of these phenotypes may point to mechanistic overlap with other copper metabolism disorders, e.g., aceruloplasminemia. We recommend genetic screening for *ATP7A* mutations in patients who manifest clinical symptoms of Wilson disease without mutations in *ATP7B*.
